# Benchmarking sustainable E‐commerce enterprises based on evolving customer expectations amidst COVID‐19 pandemic

**DOI:** 10.1002/bse.3172

**Published:** 2022-06-24

**Authors:** Saurabh Pratap, Sunil Kumar Jauhar, Yash Daultani, Sanjoy Kumar Paul

**Affiliations:** ^1^ Department of Mechanical Engineering Indian Institute of Technology (BHU) Varanasi Uttar Pradesh India; ^2^ Operations Management & Decision sciences Indian Institute of Management Kashipur Uttarakhand India; ^3^ Operations Management Group Indian Institute of Management Lucknow Uttar Pradesh India; ^4^ UTS Business School University of Technology Sydney Sydney Australia

**Keywords:** COVID‐19 pandemic, customer expectations, e‐commerce enterprises, fuzzy VIKOR, sustainability

## Abstract

The 2019 coronavirus disease (COVID‐19) pandemic has seriously impacted the performance of all types of businesses. It has given a tremendous structural boost to e‐commerce enterprises by forcing customers to online shopping over visiting physical stores. Moreover, customer expectations of the digital and operational capabilities of e‐commerce firms are also increasing globally. Thus, it has become crucial for an e‐commerce enterprise to reassess and realign its business practices to meet evolving customer needs and remain sustainable. This paper presents a comprehensive performance evaluation framework for e‐commerce enterprises based on evolving customer expectations due to the COVID‐19 pandemic. The framework comprises seven primary criteria, which are further divided into 25 sub‐criteria, including two sustainability factors, namely, environmental sustainability and carbon emissions. The evaluation approach is then practically demonstrated by analyzing the case of three Indian e‐commerce firms. The results are obtained using a multi‐criteria decision‐making (MCDM) method, namely, Fuzzy VIKOR, to capture the fuzziness of the inherent decision‐making problem. Further, numerical analysis is conducted to evaluate and rank various e‐commerce enterprises based on customer expectations and satisfaction benchmarks. The findings explain the most important criteria and sub‐criteria for e‐commerce businesses to ensure customer expectations along with their economic and environmental sustainability.

**List of Abbreviations**
COVID‐19: 2019 coronavirus disease
MCDM: multi‐criteria decision‐making
VIKOR: VIekriterijumsko KOmpromisno Rangiranje
TFN: triangular fuzzy number
B2C: business to customer

## 1 INTRODUCTION

On March 11, 2020, the World Health Organization (WHO) first officially declared the novel coronavirus disease a pandemic (Cucinotta & Vanelli, 2020; Özkan et al., 2021). This disease is commonly known as COVID‐19. As of May 3, 2022, the total number of COVID‐19 deaths worldwide has reached 6.24 million. Hence, governments worldwide are putting tremendous effort into COVID‐19 vaccination and managing medical facilities (Clemente‐Suárez et al., 2021).

The COVID‐19 pandemic has undoubtedly become one of the defining events of 2020. It has impacted almost all aspects of human lives, ranging from personal and professional lives, health concerns, shopping behavior, and businesses to the way customers spend their daily time (Chowdhury et al., 2021; Paul, Chowdhury, et al., 2021). Most nations have imposed restrictions and lockdowns to promote social distancing for varying durations in the last year. It has resulted in sudden work disruptions in manufacturing firms, especially those predominantly labor‐intensive except for a few essential products like petrochemicals and food (Seetharaman, 2020). Industries that could provide seamless services with digital offerings adapted their operations to work from home. Although most businesses offer a mix of product‐service offerings today, they have faced supply–demand disruptions at one or more stages in their supply chain (Verma & Gustafsson, 2020).

Given the insufficiencies of precedence and an unforeseen future, it is still impossible to fathom the real impact of COVID‐19 on businesses in total. However, while most companies are affected adversely, the pandemic has given a “huge structural boost” to “online industries” and especially “e‐commerce enterprises” globally (Press Trust of India, 2020). E‐commerce enterprises face tremendous pressures in current times that can make or break their businesses. On the one hand, their business is booming as consumers prefer digital purchases and omnichannel services across different e‐commerce services like consumer goods, education, electronics, health, medicine, entertainment, and food deliveries (Ivanov & Dolgui, 2020).

This global shift is evident from IBM's 2020 U.S. Retail Index report, which establishes that COVID‐19 has supercharged all digital things and has accelerated e‐commerce by approximately 5 years (Papagiannis, 2020). The strategic and operational challenges for e‐commerce enterprises have also increased due to ever‐high customer expectations. A customer expects everything, including competitive, high‐quality product‐service offerings, seamless shopping and payment experiences, safe and quick deliveries, simple returns and exchanges, and the best‐customized shopping experience possible. These customer expectations mean that e‐commerce businesses need to spend a lot of money on powerful digital capabilities to develop new product‐service ideas for their customers (Baig et al., 2020; Tran, 2020).

Customer satisfaction in the context of e‐commerce is a highly complex construct. Customers want a wide range of products at their doorstep with great ease, the lowest cost, high quality, and a wide variety while ordering. Besides, they also want options for returns and exchanges, buyback (electronic products) options, and the ability to purchase refurbished products with excellent customer service. Thus, it is a humongous task for every e‐commerce firm to meet these expectations in today's fast‐changing business environment. This paper aims to explore the intricacies of evolving customer expectations from e‐commerce enterprises. This study poses the following research questions (RQs) in particular.

○ **RQ 1:** What significant criteria should be selected to benchmark e‐commerce enterprises based on customer satisfaction?
○ **RQ 2:** What are the critical aspects and sub‐criteria of sustainability, especially environmental sustainability and carbon emissions?
○ **RQ 3:** How to rank the benchmarking criteria while capturing the intricacies of vague customer responses and subjective criteria?

The remainder of the paper is structured as follows. Section 2 presents the literature review in detail. Section 3 outlines the model formulation and selection of different criteria and sub‐criteria, and Section 4 presents the research methodology. Furthermore, Section 5 presents the working case of three e‐commerce firms from India. Finally, conclusions are presented in Section 6.

## 2 LITERATURE REVIEW

Numerous studies have shed light on how customer spending patterns have shifted during the COVID‐19 pandemic. For example, Baker et al. (2020) examined household spending in the United States during the recent COVID‐19 pandemic. They discovered that decreased movement resulted in reduced spending across all spending categories. As a result, the fewer people who move, the less money they spend on restaurants, groceries, and retail establishments (Sharma et al., 2020).

Consumer spending is declining, which has a detrimental effect on the economy. As a result, prices fall, deflation occurs, businesses fail, and job losses occur. If consumer spending remains stagnant for an extended period, the economy will eventually collapse (Manasseh et al., 2018). Consumer spending is the primary driver of the Gross Domestic Product (Mandel & Liebens, 2019). As a result, it is critical to encourage spending while discouraging residents from leaving their homes during COVID‐19. Consumers can achieve this through engagement with e‐commerce, which provides a safer alternative than in‐store shopping. Wu and Yang (2020) looked into a logistics problem and found that many things are good for the environment in the aviation industry.

According to Govender and Pretorius (2015), technology adoption occurs when an individual or organization uses a particular technology in response to a specific set of circumstances. The convenience and flexibility of e‐commerce and its general availability (24/7), prompt service delivery, and decreased human physical interactions contribute to e‐commerce's success as a shopping mall substitute (Sharma et al., 2020). Due to the pandemic disease's prevalence during this lockdown period, businesses should be proactive in establishing e‐commerce stores to serve their customers (Tran, 2020).

### 2.1 Pandemic's impact on the global economy

The previous pandemics, such as the 1918 Spanish flu, the 1957 Asian flu, the 1968 Hong Kong flu, and the 2002 SARS, impacted global annual growth rates and predicted a sharp but brief decline in the global economic trajectory (Tran, 2020). While COVID‐19 appears to pose a severe threat to global economic recovery, history demonstrates that the global economy will eventually recover. Each crisis, without a doubt, has a long‐term effect on the global economy.

Without the SARS pandemic, Alibaba would not have risen to become China's largest online retailer (CNBC, 2020). Similarly, the COVID‐19 pandemic creates a new opportunity for e‐commerce companies to engage with Indian consumers. On the other hand, the widespread pandemic of COVID‐19 in Europe has resulted in numerous changes to commerce, enticing residents to conduct business transactions digitally (Reply, 2020). Due to the prohibition of trade shows, online product demonstrations have grown popular. Consumers in the United Kingdom have shifted to online shopping, reducing their reliance on personal visits to supermarkets. As a result, Ocado's (a popular online supermarket) service has seen a 6% increase in demand (Reuters, 2020).

Compared to the United Kingdom's revival of online businesses after the COVID‐19 pandemic, consumer engagement with e‐commerce in India continues to be below average. Before the COVID‐19 pandemic, the Indian government implemented regulations that should have naturally facilitated e‐commerce, such as cashless transactions, advanced information technology (IT) infrastructure, and the availability of a variety of apps that enable access to products and brands. Due to the low acceptance rate of e‐commerce in India, it is critical to identify the factors that influence Indian consumers' engagement with e‐commerce during a pandemic (Sharma et al., 2020).

### 2.2 India and the prevalence of COVID‐19

On January 30, 2020, India reported its first case of coronavirus. On March 24, 2020, the Indian government declared a total closure of all recreational facilities in the country, including cinemas, restaurants, shopping malls, and parks. Additionally, disinfection exercises will be conducted in high‐traffic areas (Sharma et al., 2020).

Due to the lockdown, 1,366 million people across India's various regions and cities were confined to their homes, resulting in a decline in productive economic activity. Measures have been taken to mitigate the socio‐economic impact of the COVID‐19 pandemic (Sharma et al., 2020). For example, curfews compelled people to alter their daily routines, preparing them to be more proactive in responding to unexpected situational changes. As a result, many people's daily routines have shifted from offline to online, necessitating working from home. Since the pandemic of COVID‐19, the Indian government has taken significant steps to contain the virus's spread, including imposing an embargo and enforcing regulations prohibiting the virus's gathering via workplace suspension. Businesses have been impacted, and government agencies, schools, and universities were on lockdown (Bhatti et al., 2020).

Cinemas, parks, restaurants, and beaches have all been closed. Strict curfews have been implemented in all cities, and all types of gatherings are considered illegal, with fines levied for those who violate the government's directive. Notably, restaurants are permitted to open for business but are not allowed to provide in‐house or take‐out services. The country is now facing the second COVID wave, which has become more severe than the first wave. This also compels consumers to become more engaged with e‐commerce platforms indirectly. More and more consumers are becoming aware of e‐commerce platforms, especially for purchasing medical equipment.

### 2.3 E‐commerce shopping behaviour of customers

Electronic commerce, or e‐commerce, is a term that refers to a platform for the online sale of goods and services. If users utilize and engage with e‐commerce technology effectively, it can increase technical efficiency and utilization. In developing countries, consumer engagement with e‐commerce technology must increase (Hasan & Huda, 2013). According to Hasan et al. (2010), the e‐commerce industry plays a critical role in the economic development of any country. When people buy things online using e‐commerce, it has a significant impact on the world's tourism and travel industries (Nanehkaran, 2013).

E‐commerce and online shopping are advancing on the internet and other global online networks, establishing new habits for people to engage in online shopping, particularly in India (Bhatti et al., 2020). Since the COVID‐19 pandemic hit India, many people have been shopping from home, and their interest in online transactions has grown. This affects shopping habits; while many people still visit traditional markets or physical stores, they can now access online shopping apps. They are at a high risk of contracting the virus if they leave the house (Tran, 2020).

To date, e‐commerce engagement has seen significant improvements in the products and services offered to customers as the focus has shifted from the purchasing process to customer excellence (Bhatti et al., 2020). Customers can now communicate with customer service representatives via live chat to resolve service‐related issues. Additionally, customers can obtain additional information about a product by reading product reviews left by previous customers (Sharma et al., 2020). Some online retailers have made apps and websites that make customers want to visit and interact with them because they are fun and animated.

E‐commerce stores face stiff competition not only from other online retailers but also from brick‐and‐mortar retailers. By displaying reviews submitted by other shoppers, price comparison websites facilitate shopping and direct shoppers to online merchants with the best reputations (Montaldo, 2020). Please note that the biggest goal of e‐commerce is to engage and build relationships with its customers so that they do not switch to other platforms (Farooq et al., 2019). Saroja (2012) explains that the rise of e‐commerce engagement with consumers impacts retail companies that have to change their selling methods to compete. Price comparison sites make searching easier and help guide shoppers to online stores with the best reputations by posting reviews sent by other shoppers. There is also a forum for discussion where consumers can ask questions about the desired item (Rakuten Super Logistics, 2020).

The customer sees many advantages. First, the advantage of online shopping is the many variations available. They can search for all trademarks or all products in e‐commerce (Tran, 2020). Customers can follow the latest fashion trends without buying products from other countries, with no cost for plane tickets abroad. Suppose the product you are looking for does not exist in your own country. In that case, you can purchase it at an overseas retailer and feature cash on delivery, which interests consumers compared to payments made using credit cards (Bhowmik, 2012). The second is that customers do not need to go to the shopping malls. They can avoid crowding or finding parking spaces when shopping online (Anamika, 2020).

### 2.4 The present state of E‐commerce in India

The country's leaders are well aware of this, as the country has one of the world's fastest‐growing economies and largest consumer markets. As a result, they have spent the last decade attempting to diversify the economy (Sharma et al., 2020). The government has sought to reroute the economy through information and communications technology (ICT), particularly in this digital age (Bhatti et al., 2020). It is critical to understand that promoting e‐commerce in India is inextricably linked to the ease of online payment. As a result, in 2009, the Reserve Bank of India established the National Payments Corporation of India (NPCI) system to aid the Indian economy. The Unified Payments Interface (UPI) was created to facilitate online transaction processing, collection, and bill presentation (NCPI, 2020). Electronic transactions have become the norm with the government's and Indians' support. Despite the fact that few people use e‐commerce, big businesses in India rely on information and communication technology (ICT) services to ensure that services are delivered on time and in a good way (Bhatti et al., 2020).

Due to the COVID‐19 pandemic, the nation's retail establishments have been forced to remain closed (Chowdhury et al., 2021). For other businesses, it has proven to be a once‐in‐a‐lifetime opportunity. Lockdown and social distance increased user activity after many major cities declared themselves self‐isolated. In India, users' engagement with online shopping apps has increased. According to the study, both inactive and new users of online retail apps and mobile e‐commerce apps have increased significantly. In the first week of April 2020, the number of online customers increased. The pattern is similar to that in online wholesale smartphone applications. In the second week of April 2020, it was discovered that the total number of active users, potential users, and payouts was increasing (Sharma et al., 2020). In 2020, the proportion of active online platform customers was greater in March than in January and February. Most new and active mobile app customers are acquired through websites that provide food services and facilities. For example, India, Malaysia, Taiwan, Thailand, Singapore, and Hong Kong have reported increasing online traffic to South‐East Asian food websites (Tran, 2020). Chavez et al. (2016) looked at customer‐centered green supply chain management and found out what factors led to it and how well manufacturers of cars did.

A researcher could examine the increase in demand and traffic that has occurred since the beginning of March 2020. A significant increase in the number of active and new customers was observed, and it was stated that traffic had been increasing every day since March 2020. (Sharma et al., 2020). The Indian retail market is divided into two parts: the unorganized sector, which includes 13.8 million traditional, family‐owned neighborhood stores, and the organized, less than 10% retail industry. The managed market is made up of both traditional retail stores and online stores. Despite India's thriving business‐to‐consumer (B2C) e‐commerce industry, most Indians still prefer to shop at their local retail stores. They prefer to handle and feel the items before purchasing them. People in India shop at e‐commerce B2C retailers to get free items, rebates, buy one, get one free deals, and other perks (Bhatti et al., 2020).

Nonetheless, many Indian clients are considered budget‐conscious and conservative. Delivery and customer service are frequent complaints online. Personal data are stolen due to numerous e‐retailers' inadequate IT infrastructure (Bhatti et al., 2020). In India, more FTUs (first‐time internet users) are shopping online (Sharma et al., 2020). BigBasket, a major Indian grocery chain, “We return early! We are currently enemies. No one else can access our site. Please try again later.” The unexpected response crashed. In response to the high demand, competitor Grofers stated they were trying to improve efficiency and debut their firm early. Amazon, a leading U.S. and global e‐commerce company, reports that consumers rely on it more than ever. So Amazon temporarily prioritizes products like domestic goods, packaged food, health care, hygiene, and personal protection. It will stop ordering low‐priority commodities immediately. During the coronavirus outbreak, Amazon saw an increase in orders and overtime compensation (Sharma et al., 2020). Due to the coronavirus and the government shutdown, online shoppers are stocking up (to test its spread).

Previous research has considered the role of sustainability issues such as environmental carbon emissions, the role of third‐party logistics, issues related to on‐time product delivery, product authenticity, previous experience with the product, website quality, trust, privacy, and security, and so on. Moreover, there is a lack of consideration of customer satisfaction‐related factors in an e‐commerce platform in the Indian scenario. This study focused on ranking India's three major e‐commerce websites based on seven significant criteria: (1) the product: its variety, cost, authenticity, social influence, and previous experience; (2) customer service: payment methods, delivery services, and communication‐related services (complaint resolution); (3) the media's role: promotion, search time reduction, and trust; (4) the location of the warehouse and local distribution centers, the product delivery period, the delivery cost, and the possibility of product delivery; (5) public transportation: environmental sustainability, economic utility, and carbon emissions; (6) aesthetics, usability, convenience, responsive web design, and intelligent search options are all important aspects of website design; and (7) privacy and security: data security, dependability, the delivery man's identity, and the role of logistics service providers.

## 3 MODEL FORMULATION

This research considers 7 main criteria and 25 sub‐criteria during the COVID‐19 scenario. After carefully reviewing the literature, criteria and sub‐criteria are selected and finalized after discussions with industry experts. Table 1 summarizes the criteria and sub‐criteria along with their literature support.

**TABLE 1:** Summary of criteria and sub‐criteria selected for the study

Main criteria	Symbols	Sub‐criteria	References
Product purchasing	F_1_	Satisfaction with cost	Schmutz et al. (2009), Sun et al. (2014), Kohli et al. (2004), Pratap et al. (2021), Jauhar et al. (2022)
	F_2_	Satisfaction with a variety	Liu et al. (2008), Kohli et al. (2004), Nisar and Prabhakar (2017)
	F_3_	Satisfaction with product authenticity	Schmutz et al. (2009), Kohli et al. (2004), Qiu (2018), Pratap et al. (2021)
	F_4_	Earlier satisfaction with the company	Liu et al. (2008), Schmutz et al. (2009), Kohli et al. (2004)
	F_5_	Social influence	Chin et al. (2009), Kwahk and Ge (2012), Pascual‐Miguel et al. (2015)
Customer services	F_6_	Payment options	Nisar and Prabhakar (2017), Jain et al. (2020), Silitonga et al. (2020), Raj et al. (2022)
	F_7_	Delivery services	Vakulenko et al. (2019), Wilson and Christella (2019), Murae et al. (2019)
	F_8_	Communication services (resolving complaints)	Schmitz (2016), Ong and Teh (2016), Stevens et al. (2018)
Role of media	F_9_	Promotion	Hidayanto et al. (2017), Riyanto and Renaldi (2018), Arora et al. (2019)
	F_10_	Minimize search time	Poggi et al. (2014), Hidayanto et al. (2017)
	F_11_	Trust	Azam et al. (2012), Silitonga et al. (2020)
Location of warehouse	F_12_	Period of product delivery	Wilson and Christella (2019), Murae et al. (2019), Vakulenko et al. (2019)
	F_13_	The cost associated with delivery	Murae et al. (2019), Vakulenko et al. (2019), Wilson and Christella (2019), Mogale et al. (2018), Gupta et al. (2019), Prajapati et al. (2020)
	F_14_	Possibility of product delivery	Wilson and Christella (2019), Vakulenko et al. (2019), Murae et al. (2019)
Transport sharing	F_15_	Environment sustainability	Nica (2015), Gatta et al. (2019), Oláh et al. (2019), Ignat and Chankov (2020), Paul, Moktadir, et al. (2021), Prajapati et al. (2020), Ghadge et al. (2021), Prajapati et al. (2022), Pratap et al. (2022)
	F_16_	Economic utility	Jen‐Hwa Hu et al. (2017), Wilson and Christella (2019), Oláh et al. (2019)
	F_17_	Carbon emission	Nica (2015), Jauhar and Pant (2016), Jen‐Hwa Hu et al. (2017), Oláh et al. (2019), Qin et al. (2021)
Website design	F_18_	Esthetics	Cai et al. (2008), Deng and Poole (2012), Jen‐Hwa Hu et al. (2017)
	F_19_	Usability convenience	Huang (2011), Hasan et al. (2012), Jen‐Hwa Hu et al. (2017)
	F_20_	Responsive web design	Huang (2011), Zhu et al. (2017), Jen‐Hwa Hu et al. (2017), Hung and Wang (2021)
	F_21_	Smart search options	Zhu et al. (2017), Jen‐Hwa Hu et al. (2017), Barth et al. (2019)
Privacy and security	F_22_	Data security	Huang (2011), Zhu et al. (2017), Jen‐Hwa Hu et al. (2017), Goswami and Daultani (2021), Mishra et al. (2021)
	F_23_	Reliability	Vakulenko et al. (2019), Murae et al. (2019), Wilson and Christella (2019), Breneman et al. (2022)
	F_24_	Identity of delivery man	Murae et al. (2019), Wilson and Christella (2019), Vakulenko et al. (2019)
	F_25_	Role of third‐party logistics service providers	Piplani et al. (2004), Jain et al. (2020), Jauhar et al. (2021)

### 3.1 Product purchasing

While purchasing a product during COVID‐19, a customer must consider some points which could affect the product they are purchasing from a particular website (Schmutz et al., 2009). There are a lot of essential things to think about when buying a product, like how much it costs, how many options there are, how authentic the products are, how satisfied people have been with the company and the product before, and how social media can make people buy things.

As all of us know, product cost is the most influential factor in buying a product. Customers compare prices from various websites and vendors to find the best deal. However, providing lower cost prices is not the only thing a website must provide (Sun et al., 2014). The product must be available in a wide range of options from which the customer can select based on their preferences. It is also essential for the customer to make sure that they are happy with their previous products.

### 3.2 Customer services

Generally, customers deny purchasing the company's product if they are unsatisfied with the company (Nisar & Prabhakar, 2017). Also, they influence nearby people not to buy the product from a company or vendor. Hence, it represents a bad image of the website and demotivates the company from providing products and services to its customers. This research considered sub‐criteria such as satisfaction with cost, satisfaction with a variety, satisfaction with the product authenticity, previous satisfaction with the company, and social influence under this criterion.

### 3.3 Role of media

Since the number of internet users is increasing daily, many people come across social media usage. E‐commerce companies mainly focus on promoting themselves over social media and mass media, thereby using a cheap but effective platform (Hidayanto et al., 2017). We know that the media allows the customer to link directly to the websites and buy a product they want, rather than searching for it a lot on the internet.

This advertisement approach saves the customer's search time. Sometimes, various false links are also present on social media that could corrupt customers' computer/mobile systems and acquire customers' personal and financial details (Arora et al., 2019). Hence, it is necessary to know the importance of media in affecting the customer's mindset when using e‐business websites (Riyanto & Renaldi, 2018). This research considers sub‐criteria such as promotion, minimized search time, and trust under this criterion.

### 3.4 Location of warehouse

In e‐business, location in every context is essential for the customer and the related company. The warehouses and distribution centers' locations decide the time required to supply products to the customers and the cost associated with their delivery (Vakulenko et al., 2019). Many customers want the product to be delivered within a short period, but it takes some time to get delivered due to the warehouse's long distance from the customer's location (Murae et al., 2019). This causes a sense of dissatisfaction among customers.

The customer has to pay a delivery charge for short‐distance delivery. Customer location is an equally important factor for both customers and businesses since, sometimes, the logistics company cannot deliver the product directly to the customer location (Wilson & Christella, 2019). Hence, customer location is also an essential factor in deciding delivery possibilities. This study explores the sub‐criteria of time of delivery, the cost of delivery, and whether or not the product can be delivered under this criterion.

### 3.5 Transport sharing

Many companies adopt transport sharing to deliver products to customers living in distant places and require fewer products. It has been used to acquire economic utility in providing the products (Nica, 2015). It is a crucial factor in establishing environmental sustainability. Since such activity is almost sustainable and would cause fewer harmful effects on the environment, many e‐business firms widely use it (Gatta et al., 2019). Because this research uses single vehicles instead of many, they help reduce the overall carbon emissions from vehicles, which is a critical factor in reducing environmental pollution (Oláh et al., 2019). Under this criterion, current research looks at sub‐criteria for environmental sustainability, economic value, and carbon emissions.

### 3.6 Website design

One of the most critical aspects of e‐business is its website design. As customers search through the internet for any product, they find several websites. However, they approach a particular web page following factors that capture the customer's attraction (Jen‐Hwa Hu et al., 2017). These factors include the convenience of using the website, the website's overall look, the option to search smartly on the website, responsiveness of the website, and the response to fluctuating internet speed.

When he opens the website, the first thing a customer judge is whether the webpage design is attractive or not (Cai et al., 2008). If it looks good, the customer wants to be there for some time and may order some products; otherwise, he goes through without making any purchases. Also, the website should be convenient to use. Because almost every age group uses the websites, some may find these websites inconvenient and may switch to other websites (Deng & Poole, 2012).

With the advancement of internet features, some websites offer intelligent search options for users to search for the desired product easily. The facilities provided on this platform are options, a voice search system, auto suggestions, etc. Responsive web design is also a demanding criterion (Huang, 2011). The website is designed so that it should respond instantaneously to any fluctuations in web speed. The webpage should not show any errors on a low net speed availability (Hasan et al., 2012). It could motivate the customer to move away from the website, and the business may incur a lost sales opportunity. This study explores the sub‐criteria of esthetics, usability, convenience, responsive web design, intelligent search options, and how they fit into this criterion.

### 3.7 Privacy and security

Maintaining the privacy and security of the products and customers is also an essential criterion. Securing the customer's details is essential in determining website credibility (Piplani et al., 2004). Customers focus on ensuring their banking credentials and personal information to prevent financial and emotional loss. This is why e‐business firms offer proper data security throughout the purchasing process. Reliability is also a factor that sometimes affects the customer's privacy and security (Jain et al., 2020). Merely relying on the business firm for confidentiality and security purposes is not profitable for the customer.

Customers come across a few delivery men, and it is not necessary to blindly trust them in some cases. The delivery man has the customer's essential information like contact number, office address, and home address. Hence, business firms must check their identity (Vakulenko et al., 2019). Also, for keeping the security of the product, third‐party logistics service providers are somehow responsible. Any damage to the product by these parties leads to a severe loss for both the company and the customer. Although the company's significant loss is to replace the product, the customer faces time and product unavailability (Barth et al., 2019). This study considers sub‐criteria such as data security, reliability, the identity of the delivery man, and the role of third‐party logistics service providers under this criterion.

## 4 METHODOLOGY

This paper employs a fuzzy modeling approach because fuzzy can solve vague and immeasurable quantities, especially customer satisfaction. These quantities are in linguistic form but are converted into triangular fuzzy numbers (TFN) using the linguistic‐TFN converting scale. Fuzzy hierarchy methods are a more convenient way to solve these problems. It allows us to represent the problems in a specific hierarchy, making them easier to solve for firms. Specifically, we choose Fuzzy VIKOR as a solution method, as it can deal with conflicting and non‐commensurable criteria compared to other MCDM methods, like AHP. Fuzzy VIKOR also provides a reliable solution. This method adopts the principle of calculating the distance between each alternative (an e‐commerce company) and the ideal positive solution. In fuzzy VIKOR, the ranking order achieved is a compromised solution based on the alternatives' (E‐commerce companies') closeness to the ideal solution. The fuzzy VIKOR method is explained as follows.

### 4.1 Fuzzy VIKOR method

The VIKOR technique was developed in 2004 by Tzeng and Opricovic, and this technique deals with multiple‐criteria decision‐making problems having contradictory criteria and differing units. It works on selecting and ranking several alternatives (e‐commerce companies) among its set. The ranking order achieved is a compromised solution based on the alternatives' closeness to the ideal solution. Ikram et al. (2020) implemented the Fuzzy VIKOR method to identify major factors of the Integrated Manufacturing System (IMS) and proposed a policy to improve the standards of IMS development.

Fuzzy VIKOR is a method used to solve problems associated with multiple fuzzy criteria. It can solve such issues where weights and criteria are both available in fuzzy sets. These fuzzy sets are used in situations where values are vague and imprecise. In fuzzy VIKOR, it accepts all the weights and ratings in the form of linguistic variables. These linguistic variables allow the responders and decision‐makers to compare the criteria and alternatives (e‐commerce companies). The variables are then replaced by fuzzy numbers that compare the criteria. The linguistic information proposed by Wan et al. (2021) is referred to understanding the fuzzy numbers.

This study investigates the operations of e‐commerce websites during the COVID‐19 pandemic and identifies some major essential factors associated with customer satisfaction. For this purpose, 7 major criteria and 25 sub‐criteria important for customer satisfaction were identified. A survey was administered to 170 academics, industry experts, and researchers, and their responses were recorded. These responses were then used to evaluate the alternatives using the Fuzzy VIKOR method. Based on defuzzified values, the Q, S, and R values have been determined and ranked. Because of the nature of the problem, a method with multiple fuzzy criteria was required, which can deal with the fuzzy sets in both the weights and criteria. As a result, fuzzy VIKOR has been used where the values are ambiguous and imprecise. This method can help solve the problem more effectively and produce better results.

#### 4.1.1 Calculation of fuzzy importance weights of various criterions

All the criteria and sub‐criteria related to the current study are not equally important. It is necessary to calculate the fuzzy importance weights of the sub‐criteria. For this, we use the formula given below.

(1) $$p_{i}=\frac{1}{n}\left[p_{i}^{1}+p_{i}^{2}+---+p_{i}^{n}\right],$$

where $p_{i}$ is the fuzzy importance weight of the sub‐criterion Fi and $p_{i}^{n}$ is the fuzzy importance weight of criterion Fi responded by *n*th responder, and also, $p_{i}^{n}=\left(a_{i}^{n},b_{i}^{n},c_{i}^{n}\right)$.

#### 4.1.2 Construction of decision matrix

The responders rated the criteria relating to the fulfillment of customer satisfaction. However, their response depends on individual experience. They are expected to vary according to the customer's needs, choices, fulfillment, etc. Hence, it is necessary to aggregate the responses achieved by several users to synthesize the obtained response values. The formula used to find the average of acquired responses is

(2) $$\begin{array}{l} x_{\mathrm{ij}}=\frac{1}{n}\left[{x^{1}}_{\mathrm{ij}}\;⊕\;x_{\mathrm{ij}}^{2}\;⊕---⊕\;x_{\mathrm{ij}}^{n}\right],\\ {x^{n}}_{\mathrm{ij}}=\left({a^{n}}_{\mathrm{ij}},{b^{n}}_{\mathrm{ij}},{c^{n}}_{\mathrm{ij}}\right). \end{array}$$

#### 4.1.3 Identify the best fuzzy value and worst fuzzy value

The best fuzzy value is $e_{i}^{*}=\left(a_{i}^{*},b_{i}^{*},c_{i}^{*}\right)$, and worst fuzzy value is $e_{i}^{o}=\left(a_{i}^{o},b_{i}^{o},c_{i}^{o}\right)$:

(3) $$e_{i}^{*}=\max_{j}\;x_{\mathrm{ij}}\;\text{and}\;e_{i}^{o}=\min_{j}\;x_{\mathrm{ij}}\;\text{for}\;\mathrm{all}\;i\in P,$$

(4) $$e_{i}^{o}=\min_{j}\;x_{\mathrm{ij}}\;\text{and}\;e_{i}^{o}=\max_{j}\;x_{\mathrm{ij}}\;\text{for}\;\mathrm{all}\;i\in C.$$

*P* is related to benefit criteria in the above conditions, and *C* is related to cost measures.

#### 4.1.4 Calculation of normalized fuzzy difference values

The fuzzy difference value is the difference between the aggregate fuzzy values and the best fuzzy values or worst fuzzy values.

(5) $$d_{\mathrm{ij}}=\frac{\left(e_{i}^{*}-x_{\mathrm{ij}}\right)}{\left(c_{i}^{*}-a_{i}^{o}\right)}\;\text{for}\;\mathrm{all}\;i\in P,$$

(6) $$d_{\mathrm{ij}}=\frac{\left(x_{\mathrm{ij}}-e_{i}^{*}\right)}{\left(c_{i}^{o}-a_{i}^{*}\right)}\;\text{for}\;\mathrm{all}\;i\in C.$$

*P* is related to benefit criteria in the above conditions, and *C* is related to cost measures.

#### 4.1.5 Calculation of the values of *S*_*j*_ and *R*_*j*_

This step will calculate the value *S*_*j*_, which is the separation of alternative (E‐commerce company) *A*_*j*_ from the best fuzzy value and the separation of alternative (E‐commerce company) *A*_*j*_ from the worst fuzzy value. The values of *S*_*j*_ and *R*_*j*_ are estimated using the following equations:

(7) $$S_{j}=\sum_{i=1}^{k}\left(p_{i}\;⊗\;d_{\mathrm{ij}}\right),$$

(8) $$R_{j}=\max_{i}\left(p_{i}\;⊗\;d_{\mathrm{ij}}\right).$$

Here, *S*_*j*_ is the fuzzy weighted sum, denoting *A*_*j*_'s separation distance from the best fuzzy value. Similarly, *R*_*j*_ is the MAX fuzzy operator, representing *A*_*j*_'s separation distance from the worst fuzzy value. *S*_*j*_ and *R*_*j*_ can be further expanded as $S_{j}=\left(S_{j}^{a},S_{j}^{b},S_{j}^{c}\right)$ and $R_{j}=\left(R_{j}^{a},R_{j}^{b},R_{j}^{c}\right)$.

#### 4.1.6 Calculation of the values of *Q*_*j*_

The values of *Q*_*j*_ can be calculated using the following equation:

(9) $$Q_{j}=v\frac{\left(S_{j}-S^{*}\right)}{\left(S^{\mathrm{oc}}-S^{*a}\right)}+\left(1-v\right)\frac{\left(R_{j}-R^{*}\right)}{\left(R^{\mathrm{oc}}-R^{*a}\right)},$$

where $S^{*}=\min_{j}S_{j}$, $S^{\mathrm{oc}}=\max_{j}S_{j}^{u}$, $R^{*}=\min_{j}R_{j}$, $R^{\mathrm{oc}}=\max_{j}R_{j}^{u}$, and *V* = *n* + 1/2*n*, where *v* is the weight of most of the criteria. $S^{*}$ and $R^{*}$ are the best values among *S* and *R*, respectively.

Table 2 provides the linguistics variables used to represent the level of customer satisfaction and the corresponding Triangular Fuzzy Number (TFN) associated with them. Table 3 provides the linguistics variables used to describe the importance of the main criteria and sub‐criteria and the corresponding Triangular Fuzzy Number (TFN) associated with them.

**TABLE 2:** Linguistic variables and TFN for representing customer satisfaction

Linguistic variable	Scale of TFNs
Very poor	(0, 1, 3)
Poor	(1, 3, 5)
Medium	(3, 5, 7)
Good	(5, 7, 9)
Very good	(7, 9, 10)

**TABLE 3:** Linguistic value and TFN for the importance of weight measurement

Linguistic value	Triangular fuzzy number
Very low	(0, 0, 0.2)
Low	(0, 0.2, 0.4)
Fairly low	(0.2, 0.4, 0.6)
Fairly high	(0.4, 0.6, 0.8)
High	(0.6, 0.8, 1)
Very high	(0.8, 1, 1)

## 5 RESULTS AND DISCUSSIONS

Three major e‐business companies in India have been selected to analyze the results. The aim is to find the best e‐business website that satisfies the customer. Due to non‐disclosure agreements, the names of these companies are not disclosed here. The study was done considering 7 main criteria, including 25 sub‐criteria. This research surveyed 170 experts, including 95 academicians, 52 industry personnel, and 23 researchers, to observe customer satisfaction. They have also responded to the importance of the weight of the criteria.

5.1

#### 5.1.1 Calculation of necessary weights of the considered criteria

Since 25 sub‐criteria exist in the current study, each of them has a different impact on customer satisfaction. Hence, it is necessary to know the important weight of each sub‐criterion. This study will look at the responses of experts from different groups and use the correct method to show how important weight is. Table 4 provides the weight for each sub‐criteria in terms of VIKOR weight, local weight, and global weight.

1. About 170 academicians, industry experts, and researchers gave their views on the importance of the sub‐criterions. All these responses were in the form of linguistic variables. They were further converted into fuzzy triangular numbers. Using Equation (1), we convert the obtained TFN values into aggregated TFN.
2. In this step, defuzzification takes each TFN value related to the evaluator's response on sub‐criterion importance weightage, as obtained in the above step. We will also calculate the local weight and global weight of all the criteria. An illustrative example of this calculation is shown below.

Fuzzy importance weight of sub‐criterion (*F*_11_):

(10) $$\begin{array}{l} \begin{array}{rcl} \left(w_{11}\right) & = & \frac{1}{170}\left[\left(0,0,0.2\right)\;⊕\;\left(0.8,0.8,1\right)\;⊕\cdot \cdot \cdots ⊕\;\left(0,0,0.2\right)\;⊕\;\left(0.8,0.8,1\right)\right]\\ & = & \left(0.385,0.471,0.642\right) \end{array}\\ \begin{array}{rcl} \mathrm{BNP}\left(w_{11}\right) & = & \frac{\left[\left(c_{11}-a_{11}\right)+\left(b_{11}-a_{11}\right)\right]}{3}+a_{11}\\ & = & \frac{\left[\left(0.642-0.385\right)+\left(0.471-0.385\right)\right]}{3}+0.385=0.490. \end{array} \end{array}$$

**TABLE 4:** Local weights and global weights

SF	VIKOR weight	Local weight	Global weight	SF	VIKOR weight	Local weight	Global weight
F_1_	(0.385, 0.471, 0.642)	0.49	0.032	F_14_	(0.5, 0.614, 0.785)	0.633	0.041
F_2_	(0.4, 0.542, 0.742)	0.561	0.036	F_15_	(0.5, 0.671, 7.85)	0.652	0.042
F_3_	(0.728, 0.9, 0.957)	0.859	0.055	F_16_	(0.471, 0.642, 7.857)	0.632	0.041
F_4_	(0.5, 0.614, 0.757)	0.621	0.04	F_17_	(0.657, 0.771, 0.914)	0.78	0.05
F_5_	(0.128, 0.3, 0.5)	0.309	0.02	F_18_	(0.357, 0.528, 0.7)	0.528	0.034
F_6_	(0.614, 0.7, 0.871)	0.728	0.047	F_19_	(0.4, 0.571, 0.742)	0.571	0.037
F_7_	(0.5, 0.642, 0.785)	0.642	0.041	F_20_	(0.614, 0.728, 0.871)	0.737	0.048
F_8_	(0.385, 0.557, 0.7)	0.547	0.035	F_21_	(0.471, 0.614, 0.785)	0.623	0.04
F_9_	(0.5, 0.585, 0.785)	0.623	0.04	F_22_	(0.614, 0.814, 0.871)	0.766	0.049
F_10_	(0.085, 0.257, 0.457)	0.264	0.017	F_23_	(0.728, 0.9, 0.957)	0.861	0.056
F_11_	(0.385, 0.5, 0.7)	0.528	0.034	F_24_	(0.542, 0.628, 0.8)	0.656	0.042
F_12_	(0.614, 0.7, 0.871)	0.728	0.047	F_25_	(0.4, 0.542, 0.742)	0.561	0.036
F_13_	(0.428, 0.514, 0.714)	0.552	0.036				

Global weight (BNP) value of sub‐criterion $F_{11}$:

$$=\frac{0.490}{\left(0.490\;⊕\;0.561\;⊕\;0.859\;⊕\;\cdot \cdot \cdots ⊕\;0.561\right)}=0.031.$$

The local weight of the criterion is

$$\left(0.490\;⊕\;0.561\;⊕\;0.859\;⊕\cdot \cdot \cdots ⊕\;0.561\right)=15.478.$$

Table 4 illustrates the weight for each sub‐criterion in terms of VIKOR weight, local weight, and global weight.

#### 5.1.2 Estimation of the performance rating matrix

As shown in Table 2, current research uses linguistic variables to decide customer satisfaction regarding these three e‐business websites based on the mentioned criteria. Since each respondent has their own preferences, this research aggregates their preferences using Equation (2). The aggregate TFN set for customer satisfaction concerning each sub‐criterion and alternative (E‐commerce company) is shown in Table 5.

**TABLE 5:** Aggregate TFN set for customer satisfaction

Sub‐criteria	E‐commerce company 1	E‐commerce company 2	E‐commerce company 3
F_11_	(3.866, 5.80, 7.60)	(3.933, 5.933, 7.8)	(3.466, 5.4, 7.333)
F_12_	(3, 4.866, 6.866)	(3.933, 5.933, 7.733)	(4.066, 6.066, 8.066)
F_13_	(4.40, 6.333, 8.00)	(4.933, 6.866, 8.333)	(5.333, 7.266, 8.80)
F_14_	(4.733, 6.733, 8.40)	(4.333, 6.333, 8.20)	(3.733, 5.666, 7.533)
F_15_	(3.066, 5.0, 7.0)	(2.20, 4.066, 6.066)	(2.066, 3.933, 5.933)
F_21_	(4.466, 6.466, 8.133)	(3.133, 5, 6.933)	(3.933, 5.933, 7.733)
F_22_	(4.066, 6.066, 8.066)	(4.733, 6.733, 8.40)	(4.266, 6.20, 8)
F_23_	(5.266, 7.266, 8.866)	(4.066, 6.066, 7.80)	(3.983, 5.923, 7.683)
F_31_	(4.40, 6.333, 8.133)	(3.333, 5.266, 7.133)	(4.2, 6.2, 8.066)
F_32_	(3.066, 4.866, 6.733)	(2.933, 4.733, 6.533)	(2.066, 3.933, 5.866)
F_33_	(3.466, 5.40, 7.20)	(3, 5, 6.933)	(3.466, 5.40, 7.266)
F_41_	(3.933, 5.933, 7.80)	(3.066, 5, 6.866)	(3.333, 5.266, 7.20)
F_42_	(2.733, 4.60, 6.60)	(2.266, 4.2, 6.133)	(3.733, 5.666, 7.466)
F_43_	(3.466, 5.40, 7.333)	(2.866, 4.733, 6.60)	(3.80, 5.80, 7.733)
F_51_	(4.333, 6.333, 8.066)	(4.466, 6.466, 8.20)	(3.60, 5.533, 7.333)
F_52_	(4.20, 6.066, 7.80)	(4.333, 6.333, 8.20)	(4.066, 6.066, 7.866)
F_53_	(5.266, 7.266, 8.80)	(5.40, 7.40, 9)	(5, 7, 8.6)
F_61_	(3.733, 5.666, 7.60)	(3.6, 5.53, 7.4)	(4, 5.933, 7.80)
F_62_	(4.866, 6.866, 8.60)	(4.133, 6.066, 7.8)	(4.333, 6.333, 8.066)
F_63_	(4.20, 6.066, 7.866)	(4.133, 6.066, 7.866)	(4.733, 6.733, 8.533)
F_64_	(5.00, 7.00, 8.60)	(3.866, 5.80, 7.666)	(4.20, 6.20, 8.066)
F_71_	(5.533, 7.533, 9)	(5.20, 7.133, 8.666)	(5.266, 7.266, 8.866)
F_72_	(4.266, 6.20, 7.933)	(5.533, 7.533, 9.066)	(5.40, 7.40, 8.933)
F_73_	(4.0, 5.933, 7.60)	(4.60, 6.46, 8)	(4.266, 6.20, 7.933)
F_74_	(3.533, 5.40, 7.266)	(4.533, 6.466, 8.066)	(3.266, 5.266, 7.20)

#### 5.1.3 Fuzzy best value and fuzzy worst value

Current research evaluates the best and worst fuzzy values among the three alternatives (E‐commerce companies) for all the sub‐criterions in Table 6. These values are calculated by using Equations (3) and (4). Table 6 illustrates the best fuzzy TFN's and worst fuzzy TFN's among the three alternatives (E‐commerce companies) for all the 25 sub‐criteria.

**TABLE 6:** Best fuzzy alternative and worst fuzzy alternative for all sub‐criterion

Sub‐criteria	Fuzzy best	Fuzzy worst	Sub criterion	Fuzzy best	Fuzzy worst
F_1_	(3.933, 5.933, 7.8)	(3.466, 5.4, 7.333)	F_14_	(3.80, 5.80, 7.733)	(2.866, 4.733, 6.60)
F_2_	(4.066, 6.066, 8.066)	(3, 4.866, 6.866)	F_15_	(4.466, 6.466, 8.20)	(3.60, 5.533, 7.333)
F_3_	(5.333, 7.266, 8.80)	(4.40, 6.333, 8.00)	F_16_	(4.333, 6.333, 8.20)	(4.066, 6.066, 7.866)
F_4_	(4.733, 6.733, 8.40)	(3.733, 5.666, 7.533)	F_17_	(5.40, 7.40, 9)	(5, 7, 8.6)
F_5_	(3.066, 5.0, 7.0)	(2.066, 3.933, 5.933)	F_18_	(4, 5.933, 7.80)	(3.6, 5.53, 7.4)
F_6_	(4.466, 6.466, 8.133)	(3.133, 5, 6.933)	F_19_	(4.866, 6.866, 8.60)	(4.133, 6.066, 7.8)
F_7_	(4.733, 6.733, 8.40)	(4.066, 6.066, 8.066)	F_20_	(4.733, 6.733, 8.533)	(4.133, 6.066, 7.866)
F_8_	(5.266, 7.266, 8.866)	(3.933, 5.933, 7.73)	F_21_	(5.00, 7.00, 8.60)	(3.866, 5.80, 7.666)
F_9_	(4.40, 6.333, 8.133)	(3.333, 5.266, 7.133)	F_22_	(5.533, 7.533, 9)	(5.20, 7.133, 8.666)
F_10_	(3.066, 4.866, 6.733)	(2.066, 3.933, 5.866)	F_23_	(5.533, 7.533, 9.066)	(4.266, 6.20, 7.933)
F_11_	(3.60, 5.533, 7.40)	(3, 5, 6.933)	F_24_	(4.60, 6.46, 8)	(4.0, 5.933, 7.60)
F_12_	(3.933, 5.933, 7.80)	(3.066, 5, 6.866)	F_25_	(4.533, 6.466, 8.066)	(3.266, 5.266, 7.20)
F_13_	(3.733, 5.666, 7.466)	(2.266, 4.2, 6.133)			

#### 5.1.4 Calculation of normalized fuzzy difference values

To calculate the normalized fuzzy difference, Equations (5) and (6) are used. These values are shown in Table 7. An example of d_11_ is given below, which is an illustration of how Table 7 is formed.

$$\begin{array}{l} \begin{array}{rcl} d_{11} & = & \frac{\left[\left(3.933,5.933,7.8\right)-\left(3.866,5.80,7.60\right)\right]}{\left(7.8-3.466\right)}\\ & = & \frac{\left[\left(3.933-7.60\right),\left(5.933-5.80\right),\left(7.8-3.866\right)\right]}{4.334} \end{array}\\ d_{11}=\left(-0.846,0.03,0.907\right). \end{array}$$

**TABLE 7:** Normalized fuzzy difference values

Criterion	E‐commerce company 1	E‐commerce company 2	E‐commerce company 3
F_1_	(−0.846, 0.03, 0.907)	(−0.892, 0, 0.892)	(−0.784, 0.123, 1)
F_2_	(−0.552, 0.236, 1)	(−0.724, 0.026, 0.816)	(−0.790, 0, 0.790)
F_3_	(−0.606, 0.212, 1)	(−0.682, 0.091, 0.879)	(−0.788, 0, 0.788)
F_4_	(−0.785, 0, 0.785)	(−0.743, 0.086, 0.871)	(0.600, 0.229, 1)
F_5_	(−0.797, 0, 0.797)	(−0.608, 0.189, 0.973)	(−0.581, 0.216, 1)
F_6_	(−0.733, 0, 0.733)	(−0.493, 0.293, 1)	(−0.653, 0.107,0.840)
F_7_	(−0.769, 0.153, 1)	(−0.846, 0, 0.846)	(−0754, 0.123, 0.954)
F_8_	(−0.729, 0, 0.729)	(−0.514, 0.243, 0.973)	(−0.499, 0.270, 1)
F_9_	(−0.777, 0, 0.777)	(−0.569, 0.222, 1.000)	(−0.764, 0.028, 0.819)
F_10_	(−0.785, 0, 0.785)	(−0.743, 0.028, 0.814)	(−0.600, 0.200, 1)
F_11_	(−0.818, 0.03, 0.894)	(−0.758, 0.121, 1.000)	(−0.864, 0, 0.864)
F_12_	(−0.816, 0, 0.816)	(−0.620, 0.197, 1.000)	(−0.690, 0.141, 0.944)
F_13_	(−0.515, 0.205, 0.910)	(−0.462, 0.282, 1.000)	(−0.718, 0, 0.718)
F_14_	(−0.725, 0.082, 0.877)	(−0.575, 0.219, 1.000)	(−0.808, 0, 0.808)
F_15_	(−0.782, 0.028, 0.841)	(−0.812, 0, 0.812)	(−0.710, 0.203, 1)
F_16_	(−0.838, 0.064, 0.968)	(−0.935, 0, 0.935)	(−0.855, 0.065, 1)
F_17_	(−0.85, 0.033, 0.934)	(−0.900, 0, 0.900)	(−0.800, 0.100, 1)
F_18_	(−0.857, 0.063, 0.968)	(−0.810, 0.096, 1.000)	(−0.905, 0, 0.905)
F_19_	(−0.835, 0, 0.835)	(−0.657, 0.179, 1)	(−0.716, 0.119, 0.955)
F_20_	(−0.712, 0.151, 0.985)	(−0.712, 0.152, 1)	(−0.864, 0, 0.864)
F_21_	(−0.760, 0, 0.760)	(−0.563, 0.253, 1)	(−0.648, 0.169, 0.929)
F_22_	(−0.912, 0, 0.912)	(−0.824, 0.105, 1)	(−0.877, 0.070, 0.983)
F_23_	(−0.5, 0.277, 1.000)	(−0.736, 0, 0.736)	(−0.708, 0.028, 0.764)
F_24_	(−0.75, 0.133, 1.000)	(−0.850, 0, 0.850)	(−0.833, 0.067, 0.934)
F_25_	(−0.569, 0.222, 0.944)	(−0.736, 0, 0.736)	(−0.556, 0.250, 1)

#### 5.1.5 Calculation of the values of *S*_*j*_ and *R*_*j*_

This research used Equations (7) and (8) to find the values of *S*_*j*_ and *R*_*j*_, respectively. These values show the deviation of these alternatives from fuzzy best values and fuzzy worst values. They are mentioned in Table 8, and it describes the values of ( $p_{i}\;⊗$ $d_{\mathrm{ij}}$), *R*_*j*_, and *S*_*j*_ for all the sub‐criterion and alternatives.

**TABLE 8:** Values of *Sj* and *R*_*j*_ for all the criteria

Criterion	E‐commerce company 1	E‐commerce company 2	E‐commerce company 3
F_1_	(−0.326, 0.014, 0.583)	(−0.344, 0, 0.573)	(−0.302, 0.058, 0.642)
F_2_	(−0.221, 0.128, 0.742)	(−0.290, 0.014, 0.605)	(−0.316, 0, 0.586)
F_3_	(−0.441, 0.191, 0.957)	(−0.496, 0.082, 0.841)	(−0.574, 0, 0.754)
F_4_	(−0.393, 0, 0.595)	(−0.371, 0.053, 0.660)	(−0.300, 0.140, 0.757)
F_5_	(−0.102, 0, 0.399)	(−0.078, 0.057, 0.486)	(−0.074, 0.065, 0.500)
F_6_	(−0.450, 0, 0.639)	(−0.303, 0.205, 0.871)	(−0.401, 0.075, 0.732)
F_7_	(−0.385, 0.099, 0.785)	(−0.423, 0, 0.664)	(−0.377, 0.079, 0.749)
F_8_	(−0.281, 0, 0.511)	(−0.198, 0.135, 0.681)	(−0.192, 0.151, 0.700)
F_9_	(0.389, 0, 0.611)	(−0.285, 0.130, 0.785)	(−0.382, 0.016, 0.643)
F_10_	(−0.067, 0, 0.359)	(−0.063, 0.007, 0.372)	(−0.051, 0.051, 0.457)
F_11_	(−0.315, 0.015, 0.626)	(−0.292, 0.061, 0.700)	(−0.333, 0, 0.605)
F_12_	(−0.502, 0, 0.711)	(−0.380, 0.138, 0.871)	(−0.424, 0.099, 0.822)
F_13_	(−0.236, 0.105, 0.650)	(−0.198, 0.145, 0.714)	(−0.307, 0, 0.513)
F_14_	(−0.363, 0.050, 0.688)	(−0.288, 0.135, 0.785)	(−0.404, 0, 0.634)
F_15_	(−0.391, 0.019, 0.660)	(−0.406, 0, 0.637)	(−0.355, 0.136, 0.785)
F_16_	(−0.395, 0.041, 0.760)	(−0.441, 0, 0.734)	(−0.403, 0.041, 0.785)
F_17_	(−0.558, 0.026, 0.853)	(−0.591, 0, 0.823)	(−0.526, 0.077, 0.914)
F_18_	(−0.306, 0.034, 0.678)	(−0.289, 0.051, 0.700)	(−0.323, 0, 0.633)
F_19_	(−0.334, 0, 0.620)	(−0.263, 0.102, 0.742)	(−0.287, 0.068, 0.709)
F_20_	(−0.437, 0.110, 0.858)	(−0.437, 0.110, 0.871)	(−0.530, 0, 0.752)
F_21_	(−0.358, 0, 0.597)	(−0.265, 0.156, 0.785)	(−0.305, 0.104, 0.730)
F_22_	(−0.560, 0, 0.795)	(−0.506, 0.086, 0.871)	(−0.539, 0.057, 0.856)
F_23_	(−0.364, 0.250, 0.957)	(−0.536, 0, 0.704)	(−0.516, 0.025, 0.731)
F_24_	(−0.407, 0.084, 0.800)	(−0.461, 0, 0.680)	(−0.452, 0.042, 0.747)
F_25_	(−0.228, 0.120, 0.701)	(−0.294, 0, 0.546)	(−0.222, 0.136, 0.742)
*S*_*j*_	(−8.809, 1.288, 17.133)	(−8.497, 1.666, 17.702)	(−8.893, 1.419, 17.476)
*R*_*j*_	(−0.067, 0.250, 0.957)	(−0.063, 0.205, 0.871)	(−0.051, 0.151, 0.856)

An illustration of this calculation is shown as follows.

$$\begin{array}{l} S_{j}=\sum_{i=1}^{k}\left(p_{i}\;⊗\;d_{\mathrm{ij}}\right)\\ S_{j}=[\left(0.385,0.471,0.642\right)\times \left(-0.846,0.03,0.907\right)\;⊕\;\\ \left(0.4,0.542,0.742\right)\times \left(-0.221,0.128,0.742\right)⊕\cdot \cdot \cdots \cdots \cdots \cdots \cdots \\ \left(0.4,0.542,0.742\right)\times \left(-0.228,0.120,0.701\right)]\\ R_{j}=\max_{i}\left(p_{i}\;⊗\;d_{\mathrm{ij}}\right). \end{array}$$

#### 5.1.6 Identifying the values S*, R*, *S* ^*oc*^, and *R* ^*oc*^

Now, this research has identified the values S*, R*, *S* ^*oc*^, and *R* ^*oc*^ as already defined in (9).

$$S^{*}=\left(-8.893,1.419,17.476\right),R^{*}=\left(-0.067,0.250,0.957\right).$$

*S* ^*oc*^ = 17.702, and *R* ^*oc*^ = 0.957.

#### 5.1.7 Calculation of the values *Q*_*je*_

Using the values obtained in the above step and putting them in Equation (9), we will calculate the value of *Q*_*j*._ The values of *Q*_*j*_ as obtained for the given alternatives are shown in Table 9, and it shows the values of *Q*_*j*_, *S*_*j*_, and *R*_*j*_ in the form of TFN for all the alternatives.

**TABLE 9:** Values of *Q*_*j*_, *S*_*j*_, and *R*_*j*_

	E‐commerce 1	E‐commerce 2	E‐commerce 3
*Q*	(−0.037, 0.048, 0.989)	(−0.033, 0.033, 0.957)	(−0.047, 0.002, 0.945)
*S*	(−8.80, 1.288, 17.132)	(−8.496, 1.666,17.702)	(−8.892, 1.419, 17.476)
*R*	(−0.066, 0.249, 0.957)	(−0.063, 0.205, 0.871)	(−0.051, 0.150, 0.855)

#### 5.1.8 Defuzzify values of *Q*_*j*_, *R*_*j*_, and *S*_*j*_

This step will convert the *Q*_*j*_, *R*_*j*_, and *S*_*j*_ values in TFN form into the best non‐fuzzy performance (BNP) values. Equation (10) will be used in this step. The defuzzified values are shown in Table 10.

**TABLE 10:** Values of defuzzified *Q*_*j*_, *S*_*j*_, and *R*_*j*_

	E‐commerce 1	E‐commerce 2	E‐commerce 3
*Q*	0.333 (3)	0.318 (2)	0.299 (1)
*S*	9.611 (1)	10.872 (3)	10.003 (2)
*R*	1.140 (3)	1.013 (2)	0.955 (1)

#### 5.1.9 Ranking the alternatives (E‐commerce Companies)

Ranking the alternatives (*E‐commerce Companies*) based on the values of *Q* and *R* in increasing order are

$$Q_{\mathrm{Alt}3}≺Q_{\mathrm{Alt}2}≺Q_{\mathrm{Alt}1},$$

$$R_{\mathrm{Alt}3}≺R_{\mathrm{Alt}2}≺R_{\mathrm{Alt}1}.$$

The above‐ranking order states that *E‐commerce Company 3* is the best among all alternatives, following *E‐commerce Companies 2* and *1* from the perspective of customer satisfaction.

### 5.2 Sensitivity analysis

The current study examines how to rank alternatives (e‐commerce websites) that meet customer satisfaction using various primary and sub‐criteria. Sensitivity analysis is performed using the Fuzzy VIKOR method to conduct various experiments on these criteria. This study conducted a sensitivity analysis to determine how well the primary criteria worked compared to the other options (Kang et al., 2016). To accomplish this, the weight of the main criterion is changed in each operation such that the maximum weight is assigned to a single main criterion and zero to the remaining criteria. Finally, the obtained data were plotted on a graph in the form of a ranking of alternatives (e‐commerce companies).

These experiments omit the weight of the sub‐criteria with the highest gross weight (refer to Table 11) associated with a single main criterion at a time. During each experiment, current research must assume that the gross weight of each criterion is zero but keep the gross weight of all other criteria the same, so this is how it works. The sensitivity analysis is summarized in Table 11.

**TABLE 11:** Sensitivity analysis

S. No.	Condition	Closeness coefficient for (A_1_, A_2_, A_3_)	Ranking of (A_1_, A_2_, A_3_)
Exp1	$w_{3}$ = 0	(0.358, 0.451, 0.616)	(3, 2, 1)
Exp2	$w_{6}$ = 0	(0.311, 0.519, 0.639)	(3, 2, 1)
Exp3	$w_{9}$ = 0	(0.330, 0.480, 0.599)	(3, 2, 1)
Exp4	$w_{12}$ = 0	(0.293, 067, 0.638)	(3, 2, 1)
Exp5	$w_{17}$ = 0	(0.328, 0.440, 0.642)	(3, 2, 1)
Exp6	$w_{20}$ = 0	(0.354, 0.477, 0.555)	(3, 2, 1)
Exp7	$w_{23}$ = 0	(0.397, 0.342, 0.565)	(2, 3, 1)

The gross weight of criteria *I* is listed in Table 11. Experiments have been conducted using only this gross weight. Initially, the gross weight of criteria F_3_ is set to zero in experiment 1. The gross weight of criteria F_6_, F_9_, F_12_, F_17_, F_20_, and F_23_ is zero in experiments 2–7. For each of the experiments discussed previously, we left the gross weight of the other criteria unchanged.

Experiments 1–6 provide the same ranking order as the base Fuzzy VIKOR method, i.e., alternative 3 (E‐commerce company 3) is ranked first, followed by alternative 2 (E‐commerce company 2) and alternative 1 (E‐commerce company 1) at positions 2 and 3, respectively. Whereas in experiment 7, a slight variation in the ranking order is observed. Alternatively, alternative 3 (E‐commerce company 3) is ranked highest for customer satisfaction, but alternative 1 (E‐commerce company 1) is ranked second, and alternative 2 (E‐commerce company 2) is ranked third. Thus, sensitivity analysis has confirmed that alternative 3 (E‐commerce company 3) is the best e‐commerce website for customer satisfaction, followed by alternative 2 (E‐commerce company 2). Additionally, alternative 1 (E‐commerce company 1) provides the lowest level of customer satisfaction.

**FIGURE 1:**
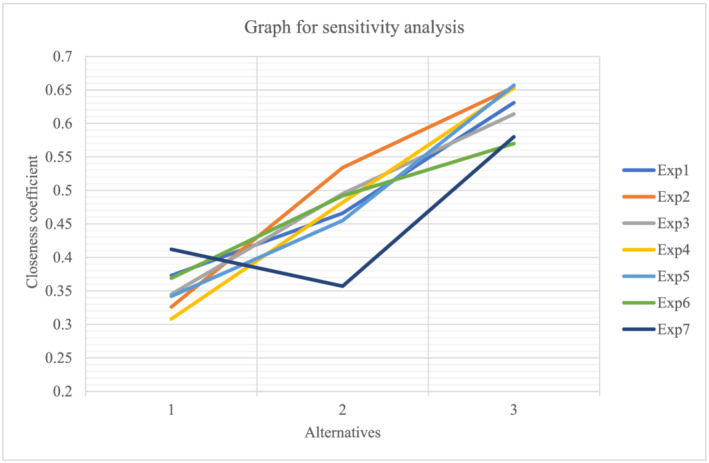
Graph for sensitivity analysis

Figure 1 depicts the relationship between the alternatives and the value of the closeness coefficient across multiple experiments. Each curve connected represents the change in the value of the closeness coefficient for the alternatives following each experiment. This way, we can see how the rankings of alternatives change after we run each of the experiments with these values of the closeness coefficient.

In experiments 1–6, for example, when gross weights for factors such as product authentication, payment option, promotion, delivery period, and carbon emission were set to 0, the value of the closeness coefficient was highest for alternative 3 and lowest for alternative 1. As a result, alternative 3 was ranked highest in these studies, followed by alternatives 1 and 2. Similarly, the gross weight allocated to the company's factor reliability in terms of privacy and security was adjusted to 0 for experiment 7. As a result, alternative 3 preserves first position, and alternatives 2 and 3 retain second and third positions, respectively.

### 5.3 Managerial insights

It is difficult to benchmark sustainable e‐commerce enterprises based on evolving customer expectations amid the COVID‐19 pandemic. Thus, in order to remain sustainable, an e‐commerce enterprise must reassess and realign its business practices in response to changing customer needs and expectations. Based on evolving customer expectations resulting from the COVID‐19 pandemic, this paper presents a comprehensive performance evaluation framework for e‐commerce enterprises. The findings indicate which performance criteria and sub‐criteria e‐commerce businesses should monitor to stay inundated and prepared for any eventuality. Based on the results of this study, managers can efficiently evaluate and benchmark their sustainable business performance.

Managers must create frameworks that serve as critical criteria for benchmarking sustainable e‐commerce enterprises. The framework is made up of several primary criteria that are further subdivided into numerous sub‐criteria. These included critical sustainability factors, such as environmental sustainability and carbon emissions, which we studied in the current study.

Managers can use an evaluation approach that has been demonstrated practically in the case of any e‐commerce firm in any country. Managers can use the current study results obtained using the Fuzzy VIKOR method. This method assists managers in capturing the fuzziness of the underlying problem. Managers can also use numerical analysis to evaluate and rank various e‐commerce companies based on customer expectations and satisfaction benchmarks.

Managers can use these findings to determine which performance criteria and sub‐criteria e‐commerce businesses should monitor to stay afloat and be prepared for any eventuality. This research could eventually help managers develop more efficient benchmarking strategies by considering several primary criteria, which are further subdivided into several sub‐criteria.

Managers can customize the framework to meet their specific requirements by including a comprehensive list of criteria and objectives. The proposed framework provides enticing opportunities for e‐commerce businesses. An individual e‐commerce business can also use the proposed framework to identify areas of weakness, allowing them to improve the overall quality and performance of their services. In summary, this study can help practitioners of e‐commerce enterprises evaluate and benchmark their businesses based on evolving customer expectations amid the COVID‐19 pandemic.

## 6 CONCLUSIONS

This article examined the relationship between evolving customer expectations and customer satisfaction using the Fuzzy VIKOR method. The conclusion of the study can be summarized as follows.

First, this research studied the operations of e‐commerce websites during the COVID‐19 pandemic and identified criteria and sub‐criteria associated with customer satisfaction. For this purpose, 7 main criteria and 25 sub‐criteria that are prominent in customer satisfaction were identified. The framework consists of two essential sustainability factors, i.e., environmental sustainability and carbon emission. A survey was conducted among 170 academicians, industry experts, and researchers, and their responses were recorded. These responses were further used for evaluating the alternatives using the MCDM technique, i.e., the Fuzzy VIKOR method. The *Q*, *S*, and *R* values have been determined and ranked based on defuzzified values. The cases of three major e‐commerce firms in India are considered to explain the working principle of the proposed solution approach.

Second, it is found that alternative 3 can fulfill customer satisfaction to a greater extent than the rest of the alternatives considered. Alternative 1 was found to be the lowest in keeping customers satisfied, whereas alternative 2 was at an intermediate rank. Third, the three components of consumer engagement—social connection, deliberate participation, and enthusiasm—can directly and positively affect evolving customer expectations. According to the current study, changing customer expectations mediate customer engagement and customer satisfaction.

The significant contributions of this study are two‐pronged. (a) It establishes seven critical criteria of prime importance for e‐commerce firms to satisfy their customers. Also, 25 sub‐criteria are analyzed in detail in the numerical example that shows their relative importance in which an e‐commerce firm has to improve its operations. (b) It details a scientific framework that e‐commerce enterprises can use to evaluate their performance on the customer satisfaction benchmark. The proposed solution framework considers that customer responses in the real world can be a little hazy at times. Further, we also present a sensitivity analysis that is useful for managers to further explore the nuances related to more complex real‐world conditions.

The study is limited as it evaluates the performance of only three top firms in India. Similar studies may shed more light on developed countries where customers are more habitual and comfortable with e‐commerce firms. Further studies may also explore the possibility of exploring customer expectations across different product categories. The study can also be extended by identifying the barriers associated with the last‐mile delivery system through various modes of transportation.

## CONFLICT OF INTEREST

The authors confirm that there is no potential conflict of interest.
